# Risk Maps for the Spread of Highly Pathogenic Avian Influenza in Poultry

**DOI:** 10.1371/journal.pcbi.0030071

**Published:** 2007-04-20

**Authors:** Gert Jan Boender, Thomas J Hagenaars, Annemarie Bouma, Gonnie Nodelijk, Armin R. W Elbers, Mart C. M de Jong, Michiel van Boven

**Affiliations:** 1 Animal Sciences Group, Wageningen University and Research Centre, Lelystad, The Netherlands; 2 Faculty of Veterinary Medicine, Department of Farm Animal Health, Utrecht University, Utrecht, The Netherlands; 3 Animal Health Service, Deventer, The Netherlands; University of Texas Austin, United States of America

## Abstract

Devastating epidemics of highly contagious animal diseases such as avian influenza, classical swine fever, and foot-and-mouth disease underline the need for improved understanding of the factors promoting the spread of these pathogens. Here the authors present a spatial analysis of the between-farm transmission of a highly pathogenic H7N7 avian influenza virus that caused a large epidemic in The Netherlands in 2003. The authors developed a method to estimate key parameters determining the spread of highly transmissible animal diseases between farms based on outbreak data. The method allows for the identification of high-risk areas for propagating spread in an epidemiologically underpinned manner. A central concept is the transmission kernel, which determines the probability of pathogen transmission from infected to uninfected farms as a function of interfarm distance. The authors show how an estimate of the transmission kernel naturally provides estimates of the critical farm density and local reproduction numbers, which allows one to evaluate the effectiveness of control strategies. For avian influenza, the analyses show that there are two poultry-dense areas in The Netherlands where epidemic spread is possible, and in which local control measures are unlikely to be able to halt an unfolding epidemic. In these regions an epidemic can only be brought to an end by the depletion of susceptible farms by infection or massive culling. The analyses provide an estimate of the spatial range over which highly pathogenic avian influenza viruses spread between farms, and emphasize that control measures aimed at controlling such outbreaks need to take into account the local density of farms.

## Author Summary

Modern approaches in the epidemiology of infectious diseases include the use of mechanistic mathematical models to analyze and predict the dynamics of disease transmission. Modelling work during the massive epidemic of foot-and-mouth-disease in 2001 in Great Britain has provided an important example of how such analyses can be performed whilst an epidemic unfolds, predicting the effectiveness of intervention efforts and thus helping to inform policymaking. In this article, we use the example of highly pathogenic avian influenza in poultry to set out a computational approach yielding risk maps for the spread of highly transmissible livestock diseases. In these risk maps, geographic areas are identified in which a given intervention strategy fails to control the spread of the disease between farms. Using the epidemiological data of a large avian influenza epidemic in The Netherlands in 2003, this approach yields an estimate of the distance-dependent transmission probability. From this transmission probability, the transmission potential is calculated for all farms in The Netherlands, leading to the identification of two high-risk areas, defined as clusters of farms with transmission potential exceeding unity. The risk map concept is an instrument suitable for analyses of epidemic control options both during crisis and in peacetime.

## Introduction

Outbreaks of highly contagious animal infections such as foot-and-mouth disease, classical swine fever, and highly pathogenic avian influenza traditionally have been and continue to be important loss factors in production animals throughout the world. In recent years, several large epidemics have occurred with serious socioeconomic consequences [1–3] and, in the case of highly pathogenic avian influenza viruses of the H5 and H7 subtypes, also with possible public health implications [4–7]. Improved understanding of the factors facilitating the introduction and subsequent spread of these viruses is crucial for effective control. An important common characteristic of these past epidemics is that a large fraction of farm infections occurred through local spread between nearby farms [8–14].

To explain the observed patterns of infection of highly pathogenic avian influenza virus between farms, and to be able to evaluate the potential effectiveness of control measures, we adopt a phenomenological modelling approach. Similar approaches have been used in modelling studies of the interfarm spread and the effectiveness of control measures during the foot-and-mouth epidemic in the United Kingdom in 2001 [8,9,14–18]. The present analysis allows us to produce geographic risk maps for the spread of highly pathogenic avian influenza virus between poultry farms. These risk maps are based on the calculation of a local reproduction number, and are constructed so as to apply to a given intervention strategy.

For estimation of the model parameters, we use an extensive dataset that was collected during an outbreak of a highly pathogenic H7N7 avian influenza virus in The Netherlands in 2003. Shortly after the detection of virus circulation, the Dutch authorities undertook an aggressive control strategy that consisted of an animal movement ban and enhanced biosecurity measures in the affected regions, tracing and screening of suspected flocks, and culling of infected and contiguous flocks. In all, 241 commercial flocks became infected during a period of 9 wk, and more than 30 million birds died by infection and culling.

**Figure 1:**
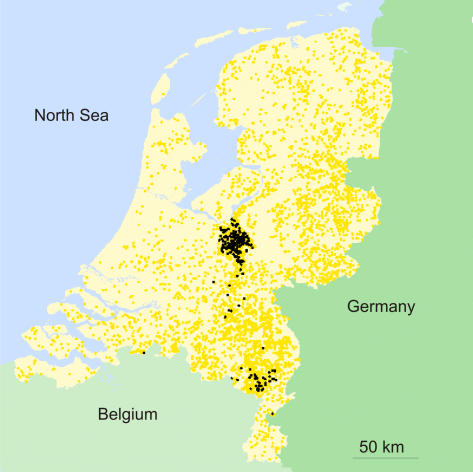
Map of The Netherlands Indicating the Physical Locations of All 5,360 Commercial Poultry Farms Farms that were infected during the 2003 epidemic of avian influenza are represented by black dots, and farms that were not infected are represented by yellow dots.

A striking characteristic of the 2003 epidemic was that most of the infected farms were confined to two areas with a high density of poultry farms. In fact, it was noted that proximity to an infected herd was a significant risk factor for acquiring infection [19]. This is illustrated by Figure 1, which shows a map of The Netherlands with the physical locations of all 5,360 commercial poultry farms. Farms that remained free of infection during the epidemic are indicated by a yellow dot, and those that were infected are represented by a black dot. The figure shows that most infections were confined to a large poultry-dense area of approximately 1,000 farms in the central part of The Netherlands (the Gelderse Vallei), and a smaller poultry-dense area of almost 400 farms in the south of The Netherlands.

**Figure 2:**
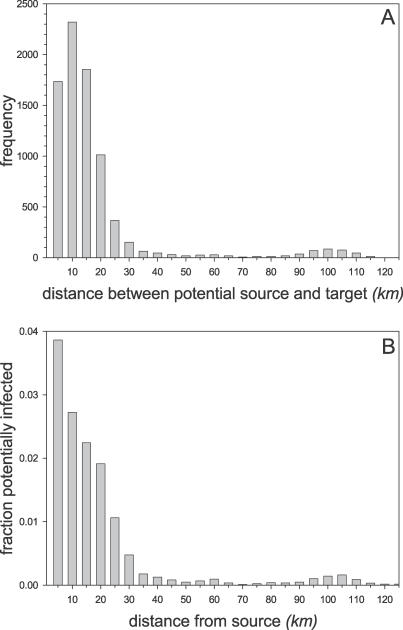
Distance Distribution of Potential Transmission Events (A) The frequency distribution of the distances of potential infection events. Notice that the majority of potential infections occur within a radius of 25 km around an infected farm. (B) The proportion of farms infected within different distance categories from a potential source farm, averaged over all possible source farms (i.e., over all farms confirmed positive during the 2003 epidemic).

As a first step to gain further insight into the spatial transmission characteristics of the 2003 epidemic, we plot in Figure 2A the between-farm distances of all potential transmission events (i.e., new infections being caused by candidate source farms). The figure shows that the majority of the potential transmission events were within a radius of 25 km around infected farms, suggesting that infection probabilities decrease with between-farm distance. This suggestion is corroborated in Figure 2B, in which we plot for each distance category the fraction of farms that are potentially infected by an infected farm, averaged over all infected farms. Notice the small hump at a distance of 90–110 km in both panels, which arises due to the distance between the two infection clusters.

Our more detailed analyses below confirm that the probability of infection decreases strongly as the distance between farms increases. In fact, the probability that a farm that is close to an infected farm (0–2 km) will be infected by that farm is 1%–2%, while farms that are further away from an infected farm (>10 km) have a probability of less than 0.05% of being infected by that farm. Our analyses also reveal that there are two high-risk poultry-dense areas in The Netherlands in which an introduction of highly pathogenic avian influenza virus is likely to cause a major epidemic. In these areas, targeted control strategies such as vaccination or culling of farms within a ring of 1–2 km around affected premises are unlikely to be effective in containing an epidemic. On the other hand, culling in a wider ring of 3–5 km may be effective, although the number of farms that has to be culled around each infected farm may become very large (>100 per infected farm).

## Methods

### Data

The analyses rely on the availability of two pieces of information. The first is the spatial locations of all farms that are at risk of infection and subsequent transmission to uninfected farms. The second is an assessment of the infection status (uninfected, infected but not yet infectious, infected and infectious, removed) of each farm during the epidemic. While the former data are relatively easy to retrieve and can be collected before or after an epidemic, the latter require a considerable effort of data collection during the epidemic. In short, as the epidemic unfolded, an attempt was undertaken to record for all infected farms the key demographic characteristics (number of barns, number of animals, type of animals, age of the animals) and data of epidemiological interest (number of dead animals per day, number of sick animals per day, food and water intake per day). By no means could all of the above information be collected for all farms, although the day at which mortality first increased and the moment of culling were reported for all farms. In our analyses, farms were assumed to be infected 6 d before the day on which mortality first increased. Upon infection, each farm then remained latently infected for 2 d, after which it was assumed to be infectious until culling. For a more detailed description of the epidemic, including detailed case reports of the first five infected premises, we refer to [19–20].

Technically, the infection data are collected in an *n* × *t*_max_ infection matrix C = (*c_ij_*), the elements of which contain the infection status of all *n* farms during the *t*_max_ days of the epidemic. For the Dutch outbreak we have *n* = 5,360 and *t*_max_ = 78 [19]. On each day, a farm is classified as susceptible (*S*), being infected on that day (*B*), infected but not infectious (*E*), infectious (*I*), or removed (*R*). Hence, *c_ij_* = *S* if farm *i* is still susceptible on day *j*, *c_ij_* = *B* if it is infected on day *j*, etc. As discussed and evaluated in [19], there is some uncertainty in the infection dates and days on which farms have become infectious. Below, we evaluate the sensitivity of our results to assumptions leading to the infection matrix C. The infection data from the infected farms can be downloaded from Dataset S1.

The farm location data take the form of a list of *n* 2-D Euclidean location vectors r*_i_* = (*x_i_*, *y_i_*). From this list we construct an *n* × *n* distance matrix D that contains the pairwise distances between farms. For reasons of privacy we cannot make the data file containing the exact locations of all farms in The Netherlands available (but see [21] for ways of constructing an approximate dataset).

### Modelling Approach and Reproduction Numbers

The model is defined on a farm level (i.e., farms are the individual units). These individual units differ by their location and infection status, and are considered identical in all other respects. This simplification (in particular, ignoring differences in farm size) is made because more detailed modelling introduces additional parameters that cannot be estimated with sufficient precision to add further insight into the spatial transmission risk. The infection matrix C discussed above specifies how each farm's status (*S*, *B*, *E*, *I*, or *R*) developed through time during the epidemic in 2003. The model assumes that the probability *p*(*r_ij_*) that an uninfected farm *j* will be infected by an infected farm *i* depends only on the infectious period *T_i_* of farm *i* and on the (Euclidean) distance *r_ij_* = |*r_i_* − *r_j_*| between the farms, and is given by *P*(*r_ij_*) = 1 − exp(−*h*(*r_ij_*)*T_i_*). The function *h*(*r_ij_*) is called the *transmission kernel,* and is defined as the infection hazard posed by farm *i* to farm *j* as a function of interfarm distance [8,9,14,18]. In the next section, we describe how the transmission kernel *h*(*r_ij_*) is estimated from the data matrices C and D.

With estimates of the transmission kernel at hand, a risk map can be constructed in the following way. At every farm location we calculate the (basic) reproduction number *R*, which equals the expected number of secondary infections caused by one infected farm in the early stages of an epidemic (i.e., before the depletion of susceptible farms starts to play a role). If *R* > 1, the pathogen is able to cause a major epidemic, while it cannot if *R* < 1. If we denote the density of farms at a distance *r* from a focal farm *i* by *ρ_i_*(*r*), then standard arguments show that the reproduction number of farm *i* is to a good approximation given by

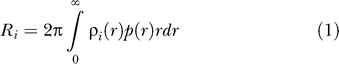
For more accurate approximations taking into account the effects of local depletion of susceptible farms we refer to (Boender GJ, Meester R, Gies TJA, de Jong MCM, unpublished data). If farm density were constant (*ρ_i_* = *ρ*), the individual reproduction numbers are identical, and the above expression simplifies to

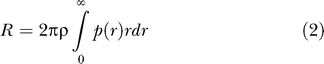
The integrand *p*(*r*)*r* in the above expression determines the contribution of farms at distance *r* to the reproduction number. Notice that the reproduction number in the above equation is proportional to the farm density. The critical farm density *ρ_c_* is given by the solution of the equation *R* = 1. Using the above result for *R*, this yields

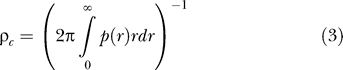
The above equations show that, under certain assumptions, the reproduction number can be translated into a farm density and vice versa. In particular, the threshold condition *R* = 1 for epidemic spread translates into a critical farm density.

The above theoretical considerations assume that farm density is constant. This is hardly ever true in practice, and we need to take into account the actual distribution of farms. For a specific distribution of farms and assuming a stochastic infectious period 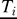 the reproduction number *R_i_* of farm *i* is given by

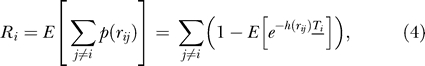
where the index runs across all farms in the population except the focal farm.

Equation 4 gives the expected number of infections caused by an infected farm in the early stages of an outbreak. For completeness, we note that not only the expected number of infections (i.e., the reproduction numbers) but also the complete offspring distribution (i.e., the distribution of the number of farms infected by a single infected farm) in the early stages of an epidemic can be determined by simulation using the elements in the summation of Equation 4. It appears that the resulting distributions are almost indistinguishable from Poisson distributions with parameters *R_i_* (unpublished data). This phenomenon can be explained theoretically if the individual farm contributions are determined by a (inhomogeneous) Poisson process. In our model, this condition would hold true if the farm locations are generated by a (inhomogeneous) Poisson process. Simulations show that for the Dutch farm data, the Poisson approximation gives an excellent description of the actual offspring distribution, even though the farm locations are highly overdispersed (unpublished data).

If the infectious periods are drawn from a parametric distribution, an explicit expression for *R_i_* can usually be obtained. For instance, if the infectious periods arise from a common gamma distribution with shape parameter *c* and scale parameter *T*/*c*, then the reproduction number of farm *i* is given by

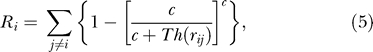
where *T* and 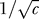 are the mean and variance of the infectious period probability distribution, respectively. Here the special cases of a fixed or exponentially distributed infectious period are obtained by letting *c* → ∞ or by taking *c* = 1, respectively.

Below, we obtain a risk map for epidemic spread by drawing a map in which all commercial poultry farms are indicated by a dot, representing those farms with *R_i_* > 1 by a red dot. This identifies high-risk areas as red areas on the map.

### Parameter Estimation

To obtain quantitative estimates for the local reproduction numbers, we need estimates of both the transmission kernel *h*(*r_ij_*) and the (distribution of the) infectious period *T_i_*.

The infectious periods at the farm level were obtained from the infection matrix C. The mean infectious period of the 241 farms that were infected was 7.47 d (95% CI = (7.2–7.8). On the basis of these data we took *T* = 7.47 and *c* = 11.1 in Equation 5.

We estimate the transmission kernel *h*(*r_ij_*) from the data matrices C and D by means of maximum likelihood [8,14,18]. In line with previous studies [9,14], we parameterised the transmission kernel using a three-parameter logistic expression:

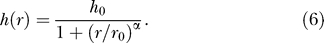



To evaluate the performance of the transmission kernel specified by Equation 6, we considered a number of alternative functions, and compared the performance of the kernel specified by Equation 6 with the alternatives on the basis of Akaike's Information Criterion (AIC) [22]. The results are discussed below.

To derive the likelihood function, we define the force of infection *λ_i_*(*t*) on a susceptible farm *i* by:

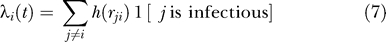
(1 represents the indicator function). The force of infection determines the probability *q_i_*(*t*) that a hitherto susceptible farm *i* is infected on day *t*,

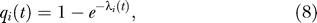
and the probability *r_i_*(*t*) that farm *i* remains uninfected up to day *t*,

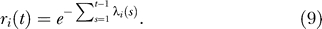



Using Equations 8–9, the likelihood function takes the following form:

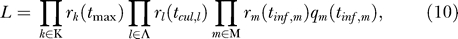
where the set K contains all farms that remained uninfected and that were not culled, Λ contains the farms that were not infected but that were culled (at times *t_cul,l_*), and M contains the farms that were infected (at times *t_inf,m_*). In practice, it is computationally more efficient to use the log-likelihood ℓ = log(*L*) instead of the likelihood *L*. Using Equations 8–10, the log-likelihood can be written in terms of the forces of infection as follows:

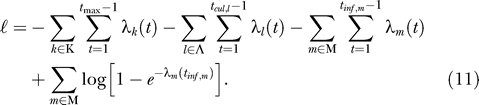



The maximum likelihood estimates of the parameters of interest (*h*_0_, *r*_0_, and *α*) are readily obtained by maximization of Equation 11. The confidence bounds of the parameters (Table 1) are calculated using profile likelihoods. The 95% confidence bounds of the transmission kernel shown in Figure 2 are calculated by determining, at any given distance, the range of values spanned by the kernel when the kernel parameters run across the 95% confidence volume (as determined by the profile likelihood). Below we comment on the sensitivity of the results with respect to the spatial range of the farms that were included in the estimation procedure.

**Table 1:**
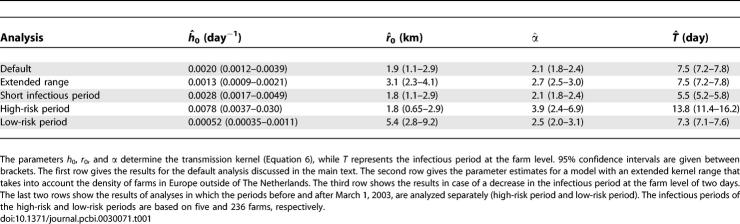
Maximum Likelihood Estimates of the Model Parameters

**Figure 3:**
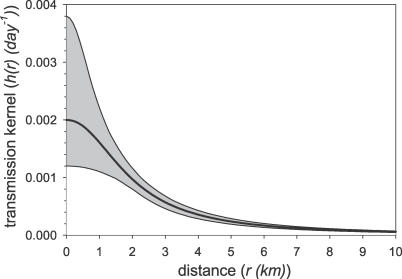
The Transmission Kernel as a Function of Interfarm Distance for the Parameter Estimates of Table 1 The 95% confidence areas of the transmission kernel are represented by the shaded area.

**Figure 4:**
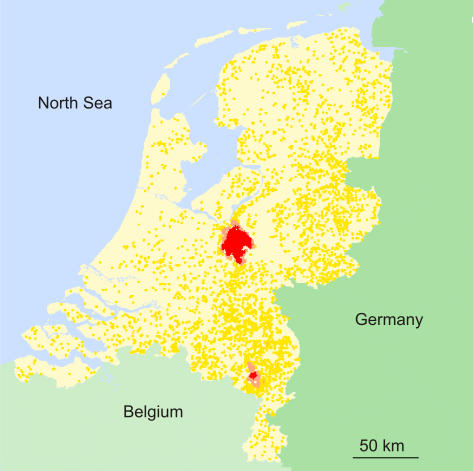
High-Risk Areas for Epidemic Spread of Avian Influenza Virus Based on the Transmission Kernel of Figure 3 See Table 1 for parameter estimates. For each farm, an individual reproduction number *R_i_* is calculated on the basis of Equation 5. Infected farms with *R_i_* < 1 infect, on average, less than one susceptible farm and pose no risk for epidemic spread (yellow dots). Infected farms with *R_i_* > 1 are expected to infect more than one susceptible farm in the early stage of an epidemic and thus constitute a risk of epidemic spread (red dots). Pink dots represent farms with *R_i_* < 1 for the maximum likelihood estimate of the transmission kernel, but with *R_i_* > 1 for the upper boundary of the 95% kernel confidence area (Figure 3). Note that most of the farms that were infected during the epidemic in The Netherlands in 2003 (Figure 1) are classified as high-risk farms.

**Figure 5:**
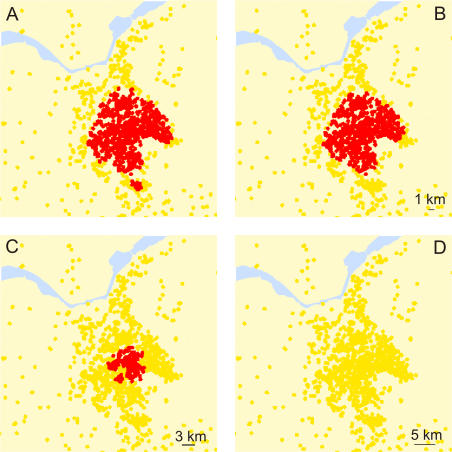
High-Risk Areas for Epidemic Spread for Various Local Culling Strategies in the Central High-Risk Area of The Netherlands (A) Results for the default scenario (no culling). (B) Results for a scenario with immediate culling of all farms within a range of 1 km around an infected farm. (C,D) Culling is carried out in a range of 3 km and 5 km around infected farms, respectively. Farms in yellow pose no risk of epidemic spread for the chosen control strategy, while farms in red constitute a risk of epidemic spread even with the control strategy in place.

Mathematica 5.2 (Wolfram, http://www.wolfram.com) was used for all data processing, modelling, and statistical analyses. Figures 2 and 3 were produced using SigmaPlot 10.0 (Systat Software, http://www.systat.com). Figures 1, 4, and 5 were also produced using Mathematica 5.2.

## Results

### High-Risk Areas for Epidemic Spread

Table 1 shows the parameter estimates of the transmission kernel describing the transmission rate from infected to uninfected farms as a function of interfarm distance, and Figure 3 displays the transmission kernel for these parameter estimates. The transmission rate decreases from an estimated 2.1 × 10^−3^ (d^−1^) in the direct neighbourhood of an infected farm to 1.6 × 10^−3^ (d^−1^) at 1 km, and to 6.1 × 10^−3^ (d^−1^) at 10 km distance. This implies that the probability that a given farm will be infected if it is at 0 km, 1 km, or 10 km from an infected farm is approximately 0.016, 0.012, and 4.6 × 10^−4^ if the infectious period is 7.5 d. Figure 3 also shows the uncertainty associated with the estimate of the transmission kernel. At short distances the uncertainty is largest because the number of datapoints is lower here as there are far fewer farms at short distances than there are at long distances.

Figure 4 shows a risk map of The Netherlands based on the kernel estimate of Figure 3. Farms in yellow are expected to produce fewer than one new infection if infected (i.e., *R_i_* < 1), while farms in red are expected to produce more than one new infection if infected (*R_i_* > 1). Farms in pink do not have a reproduction number larger than 1 for the maximum likelihood estimate of the transmission kernel, but do have a reproduction number exceeding 1 for the 95% confidence upper bound of the transmission kernel in Figure 3. Areas in which farms with *R_i_* > 1 predominate are at risk of epidemic spread, while an epidemic cannot occur in areas in which farms with *R_i_* < 1 prevail.

The analysis shows that there are two areas in The Netherlands that are at risk of a locally propagating epidemic after a virus introduction: one large area in the central part of the country comprising 913 farms (95% confidence bounds of the high-risk area: 685–1,065) and one small area in the south of 61 farms (95% confidence bounds of the high-risk area: 0–206). In those two areas the local density of poultry farms is such that an infected farm is expected to produce a substantial number of subsequent infections.

A comparison of Figure 1, which shows the farms that were actually infected during the 2003 epidemic, and Figure 4, which shows the areas (farms) that are calculated to have a high risk of epidemic spread, shows that there is a good agreement between the two. In fact, using the estimated transmission kernel, 162 of the 241 infected farms are also classified as being in a high-risk area by our method, while 79 of the infected farms were located in areas that are classified as having a low risk of epidemic spread. The correspondence improves further if one takes the 95% confidence upper bound of the transmission kernel as the basis of analysis, thereby adding a number of pink farms in Figure 4 to the high-risk area (189 infections in high-risk areas versus 52 in low-risk areas). Altogether, our classification scheme appears to work very well in general, although there remain a number of infected farms in areas that are classified as low-risk. Fortunately, and as predicted by our method, these infections in the low-density areas did not spark new outbreaks in the low-density areas.

At this point we would like to note that, in order not to miss or underestimate the size of high-risk areas close to the Dutch border, we have incorporated the poultry farms in the Belgian provinces and German administrative areas (NUTS2 administrative levels) bordering The Netherlands in our calculations (both in the kernel estimation and in the risk-map calculation). As we do not have access to location data for the farms in these regions, we have approximated the farm structure of these regions by generating random model locations on the surface of these regions according to a homogeneous Poisson point process. The total number of model locations per region was matched to Eurostat data (http://ec.europa.eu/eurostat) on the total numbers of farms by region.

To investigate the robustness the above results, we performed a suite of sensitivity analyses. We paid particular attention to the functional form of the transmission kernel, the range of farms included in the estimation procedure, the assumptions leading to the infection data matrix, and the assumed constancy of the transmission level over time. Below, we discuss each of these aspects in turn.

### Alternative Transmission Kernels

As a first step to investigate the sensitivity of the above results we considered a number of different functions of varying complexity for the transmission kernel. The performance of the different kernels was evaluated on the basis of the support received by AIC [22]. The results of the analyses are summarized in Table 2. In general, the analyses show that simpler kernels with only one or two parameters fit the data significantly worse than our default three-parameter logistic equation (i.e., Equation 6), which has by far the highest support. This indicates that we avoided overfitting the data.

**Table 2:**
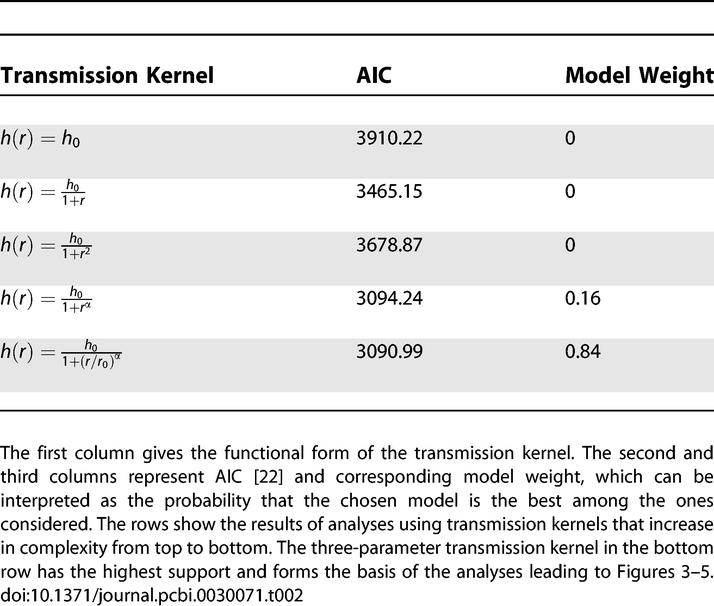
Evaluation of the Performance of Different Transmission Kernels

Table 2 shows that of the distance-based models a kernel without any distance dependence in the transmission risk (*h*(*r*) = *h*_0_) gives by far the worst fit to the data and has negligible support in comparison with models that do include some form of negative distance dependence. These results imply that the risk of transmission is not constant but decreases with interfarm distance. In particular, the results of Table 2 indicate that models that do not allow a rapid decrease of the transmission kernel at long distances perform much worse than models in which the tail of the transmission kernel quickly drops of to very low values (i.e., for which the tail is at least of the order 1/*r^α^* where *α* > 2). This implies that farms at very long distances contribute marginally to the farm reproduction numbers, even though there are many more farms at long than at short and intermediate distances.

To further investigate the sensitivity of our results, we considered alternative transmission models in which the transmission kernel does not depend on the Euclidean distance between farms, but on the distance rank of infected farms to susceptible farms, or the distance rank of susceptible farms to infected farms (see [23] for still other alternatives]). In a hypothetical situation where most of the transmission takes place through human contacts between farms, such models would be appropriate whenever there is a fixed rate at which people visit neighbouring farms but are more likely to visit nearby farms than those that are farther away. Simulations based on estimates of the rank-based transmission kernels show that these models are unable to capture the patterns of infection of the Dutch epidemic (unpublished data). In particular, these models predict that occasional infections in the low-density areas will not remain isolated but cause new clusters of epidemic spread in the low-density areas. Hence, the rank-based models predict that epidemics that are started in the high-density area in the central part of The Netherlands will ultimately spread all over The Netherlands.

### Range of Farms Included in the Kernel Estimation

Our kernel estimates are based on all commercial farms in The Netherlands. Since The Netherlands is a small country (35,000 km^2^), this implies that the kernel parameter estimates are based mainly on pairs of farms that are less than 150 km apart. To investigate the sensitivity of the results to the range of distances for which information is available, we have repeated the kernel estimation using an extended dataset in which the distribution of poultry farms outside The Netherlands was taken into account. Specifically, we approximated Europe by one-half of an annular area of inner radius of 200 km and an outer radius of 1,600 km with a uniform poultry farm density equal to the mean poultry farm density of the 24 non-Dutch European Union member states (http://ec.europa.eu/eurostat) and re-estimated the transmission kernel.

The results show that the inclusion of farms outside of The Netherlands (and the information that these had not been infected) only marginally affects the estimated local reproduction numbers, yielding risk maps that are indistinguishable from those in Figures 4 and 5 (unpublished data). However, we do find that the kernel parameter estimates are changed considerably compared with the default analysis (Table 1). This paradox arises due to neutralizing effects between the three parameter shifts. The net result of the changes in the three parameters is a mere lowering of the long-distance tail of the transmission kernel, mostly due to the higher estimated value for the parameter *α*. The kernel hardly changes at short and intermediate distance scales, which together are the dominant contributors to the local reproduction numbers.

### Uncertainty in the Temporal Course of the Epidemic

In the above analyses, we assumed perfect knowledge of the course of the epidemic in The Netherlands in 2003. There is, however, some uncertainty in the data with regard to the infection matrix C, in particular with respect to the precise moment of infection of infected farms. To investigate the sensitivity of the results to assumptions underlying the infection matrix, we carried out additional analyses in which the moment of introduction was placed 2 d later than in our default analyses (see [19] for details). The sensitivity analysis (Table 1) shows that the decrease in the infectious period by 2 d is offset by a corresponding increase in the height of the transmission kernel such that the individual farm-level reproduction numbers remain roughly the same (unpublished data). In particular, the baseline infection hazard parameter *h*_0_ increases from an estimated 0.0020 d^−1^ in our default scenario to 0.0028 d^−1^ in the additional analyses. Hence, the sensitivity analysis indicates that although the estimates of the parameters of the transmission kernel (in particular the baseline infection hazard *h*_0_) are sensitive to the moment of introduction and length of the infectious period, the risk map of Figure 4 is remarkably insensitive to the precise assumptions leading to the infection data matrix. We observed the same phenomenon in our earlier nonspatial analysis of the same epidemic [19].

### High-Risk versus Low-Risk Periods

The above analyses assume that both the infectious period at the farm level as well as the transmission kernel remained constant throughout the epidemic. This, however, is only approximately the case. Especially during the first week of the epidemic there were no or hardly any control measures in place, and the detection of infected farms was still imperfect and slow. In line with our earlier nonspatial analyses [19], we have therefore performed analyses of the period before and after relevant control measures had been put in place (i.e., 1 March 2003). The results of the analyses, shown in Table 1, illustrate that both the infectious period as well as the height of the transmission kernel at short distances (<4 km) was considerably higher in the high-risk period compared with the low-risk period. On the other hand, at long distances (>4 km) the transmission kernel was somewhat higher in the low-risk period compared with the high-risk period. This may seem counterintuitive, but it should be noted that the parameter estimates of the high-risk period were based on a fairly small number of infected farms and on a small number of days. The differences between the transmission kernels of the two analyses may seem large (as judged by the differences in the parameter estimates), but in fact the transmission kernels are quite similar at the medium-range distances (2–5 km) that contribute most to the local reproduction numbers. The risk maps that are constructed using the parameter estimates pertaining to the high-risk and low-risk periods are much like the risk map for the analysis in which the epidemic is analysed in one go. For instance, the number of high-risk farms in the central area of The Netherlands in the default analysis contained 913 farms (see above), while the number of high-risk farms in the analyses of the high-risk and low-risk periods contained 1,015 and 863 farms, respectively.

### Local Containment

Besides a movement ban and biosecurity measures, there are two potentially attractive local control measures: culling of farms in the proximity of infected premises that have a heightened risk of infection, and vaccination. We first investigate the effectiveness of rapid culling of all farms in a ring around infected farms. The effect of this measure can be described by reducing the height of the transmission kernel of the infected farm and/or the length of the infectious period of potential contact farms within the culling radius. Here, we assume that culling occurs before any infected farm in the culling ring starts spreading the infection to other farms, so that the intervention can be described by setting the transmission kernel of the infected farm to zero at distances within the culling radius. Thus, the analyses below correspond to a best-case scenario, and assume in effect that no transmission takes place from farms within the culling radius. This can probably only be achieved if ring culling is carried out within a couple of days after infection of a focal farm.

Figure 5 shows the results of the analyses for the central part of The Netherlands that was identified above as a major high-risk area. Farms that do not constitute a risk are drawn in yellow, while farms that pose a risk of epidemic spread are depicted in red. The figure shows high-risk farms in case of no culling (Figure 5A; compare with Figure 4), in case of culling in a ring of radius 1 km (Figure 5B), and in case of culling in a ring of radius 3 km or 5 km (Figure 5C and 5D). Culling of farms in a ring of 1 km does not appear to be very effective, as most farms that were classified as high-risk without culling remain high-risk with a 1-km culling strategy in place. Culling within a ring of 3 km in radius is more effective, although there are still more than 100 farms that are classified as high-risk (i.e., that are expected to produce more than one subsequent infection once infected). Culling in a radius of 5 km is fully effective in the sense that with this control strategy there are then no farms that are expected to produce more than one subsequent infection once infected. The question remains whether rapid culling of all farms in a radius of 5 km around an infected farm is feasible logistically, as the number of farms that have to be culled increases rapidly with the culling radius. In fact, for the high-risk area in the central part of The Netherlands, the average density of neighbour farms exceeds 4 farms/km^2^ so that a 5-km ring culling strategy would imply that for each infected farm more than 300 farms have to be culled within a couple of days (*π* × *r* ^2^ × *ρ* = 3.14 × 25 × 4 = 314). If the infection has spread already within this high-risk area at the time of the first detection, this implies that the majority of the approximately 913 farms in the area would need to be culled. Apart from the logistic difficulties, it would be difficult politically to decide in favor of such a massive culling policy, as the Dutch Ministry of Agriculture, Nature and Food Quality, in view of public opinion in The Netherlands, has declared it their policy to resort to emergency vaccination strategies instead.

As far as emergency vaccination around infected farms is concerned, we note the following. On the one hand, it is highly unlikely that vaccination can be effective once a highly pathogenic virus has successfully been introduced in a densely populated poultry region. The reason is that it takes at least a week to vaccinate all susceptible poultry and an additional 7–14 d before a vaccine provides effective protection against infection and subsequent transmission [24]. This time span would give the virus ample opportunity to spread throughout the area. On the other hand, vaccination is increasingly being considered as a possible tool to prevent the successful introduction of the disease in certain high-risk areas in case a highly pathogenic virus has been detected at a certain distance from the area.

## Discussion

In this paper we have presented an analysis of the spatial transmission dynamics of highly pathogenic avian influenza virus spread between farms by using an extensive dataset of a major epidemic of H7N7 avian influenza virus in The Netherlands in 2003. As the specific transmission route responsible for infection is unknown for all of the infected farms, we have adopted a phenomenological modelling approach in which we do not distinguish between different specific routes contributing to between-farm virus transmission. This allows us to obtain quantitative estimates of model transmission rate parameters that describe the transmission risk between pairs of farms as a function of distance.

We have shown how the estimation of the transmission kernel naturally leads to estimates of a local reproduction number, which allows one to map out the transmission risk geographically. In this way, two poultry-dense areas at risk of local epidemic spread are identified in The Netherlands. The local reproduction number can be interpreted as a measure of the local farm density or, more precisely, as a measure of the density of farms surrounding a farm at a given location. As a result, we may view the geographic risk map as a farm density map. In particular, the critical farm density above which epidemic spread is possible corresponds to a situation where the local reproduction number equals the threshold value 1.

The density of poultry farms happens to differ quite strongly between the high-risk areas and elsewhere in The Netherlands. For instance, while the average farm density in the two areas that were classified in our analyses as high-risk (913 farms) is about 3.8 farms/km^2^, the average density in the remainder of The Netherlands (4,447 farms) was only about 0.5 farms/km^2^. This corresponds well with Equation 3, which predicts that for the kernel estimate of Table 1 the critical farm density in a spatially homogeneous population would be 2.9 farms/km^2^. As a result of the large differences in poultry densities in The Netherlands, moderate changes in the transmission kernel have very little effect on the important features of the risk map, in particular the location and the size of the high-risk areas. Due to this insensitivity, our results are robust under variation of uncertain parameters.

By adjusting the transmission kernel, we have also produced risk maps that evaluate the effectiveness of pre-emptive ring culling around infected farms. These risk maps show that pre-emptive culling within 3 km or less is unlikely to be able to halt an unfolding epidemic in the high-risk areas. In these areas, an epidemic can only be brought to an end by the depletion of susceptible farms by infection or massive culling. Our analyses indicate that in the high-density areas in The Netherlands, ring culling is only effective (in the sense that it can halt an unfolding epidemic) if the culling radius is more than 3 km. On the other hand, our analyses also show that in the remainder of The Netherlands (i.e., the large low-density areas of Figure 3), pre-emptive culling is probably not necessary to halt the disease from spreading after a primary introduction.

An important open problem is whether culling or vaccination programmes are able to reduce the total number of animals that would die during an epidemic (by infection or culling) compared with a strategy in which only a movement ban and biosecurity measures are put in place. During the epidemic of the highly pathogenic H7N7 avian influenza virus that wreaked havoc in The Netherlands in 2003, it was decided, on the basis of the then-available epidemiological and economic information and legislative constraints, to put in place an aggressive control strategy in which culling around infected premises was an integral part. Based on the present analyses, we would expect that an introduction of a highly pathogenic avian influenza virus in one of the poultry-dense areas in The Netherlands cannot easily be contained, and probably would affect a large fraction of the farms in such a region. However, some form of preventive culling around infected premises would still pay off, as it would decrease the length and severity of the epidemic in this region, and thereby also reduce the risk of spread of the disease to other (high-density) areas.

## Supporting Information

Dataset S1 Infection Status over Time of all 241 Farms That Were Infected during the Epidemic of Highly Pathogenic H7N7 Avian Influenza Virus in The Netherlands Each line gives the infection status (*S*, *B*, *E*, *I*, *R*) per day per farm.
(72 KB TXT)

## Abbreviations

AIC: Akaike's Information Criterion
